# Elevated Temperature in Some Cases of Pernicious (Congestive) Malarial Fevers

**Published:** 1886-08-20

**Authors:** Joleph Jones

**Affiliations:** Visiting Physician of Charity Hospital, New Orleans, Louisiana; Professor of Chemistry and Clinical Medicine, Tulane University of Louisiana, etc.


### ELEVATED TEMPERATURE IN SOME CASES OF PERNICIOUS (CONGESTIVE) MALARIAL FEVERS.

By JOLEPH JONES, M. D.,

Visiting Physician of Charity Hospital, New Orleans, Louisiana; Professor of Chemistry and Clinical Medicine, Tulane University of Louisiana, etc.

It is worthy of note that many of the cases of congestive or pernicious malarial fever, and especially of the *comatose* variety, frequently manifest an elevated temperature.

The following cases will illustrate this proposition:

### CASE I.

*Comatose Malarial Fever.*—Emanuel Carneno, native of Portugal, entered ward 30, bed 448, Charity Hospital, October 13, 1874; had suffered with malarial fever for four months; pale, anæmic, sallow, dark hue; general anasarca; urine free from albumen. He appeared to be improving slowly under the action of quinine, iron, arsenic.

On the 5th of November, 1874, the patient was seized with a severe chill and became comatose. There was a rapid rise in temperature, and one and a half hours before death, whilst the patient was in a profound coma, the temperature of the axilla was 107.5°. No albumen in the urine. Died during the night of the 5th, all measures having failed to arouse the patient from the comatose state.

*Post-Mortem Examination.—Heart* and lungs normal. *Blood* deficient in blood corpuscles, and of low specific gravity, abounding in a thin serous fluid; general anasarca. *Liver* dark brown and slate color on the exterior; interior of a dark hue; deposit of dark pigment, and pigment cells within and around hepatic capillaria. Bile thick, dark and abundant, about 18,000 grains of dark bile in gall-bladder. *Kidneys* normal. *Bladder* contained high-colored urine, rich in urea, but free from albumen casts and colored blood corpuscles. *Cranium*, membranes of brain as well as the blood vessels of the brain substance greatly congested with dark blood; cortical substance of the cerebrum and cerebellum of a dark gray and chocolate color; deposits of dark pigment and of large pigment cells within and around central capillaries. Blood, urine, brain, and spinal cord congested with blood.

### CASE II.

*Pernicious Malarial Fever.*—In a similar case, which was brought into Charity Hospital in a comatose condition, the patient, although anæmic and anasarcous with enlarged spleen and liver, manifested during the early forty-eight hours of life passed in a profound coma, a temperature in the axilla of from 104° to 106°F. Immediately before death the temperature was 106°.

### CASE III.

*Pernicious Malarial Fever.*—M. Shea; age twenty-seven; entered ward 18, bed 206, Charity Hospital, November 2, 1884; comatose; passes urine and fæces in bed; jaundiced; hot, dry skin. There was marked capillary congestion; great epigastric tenderness; urine contains blood corpuscles (red), albumen, and blood casts of the tubuli uriniferi. Liver and spleen enlarged. Heart and lungs healthy. Pulse rapid and feeble, 172 beats per minute; respiration 42 per minute; temperature of axilla 103.7°. The patient continued in a comatose state and died within twelve hours after his entrance into the hospital. During this period the eyes were fixed in one position, and the pupils did not respond to light.

It was ascertained that the patient had suffered with malarial fever before entering the hospital.

*Post-Mortem Examination.*—Heart and lungs normal. Dependent portions of lungs congested. Pericardium contained about two fluid-ounces of golden yellow fluid. Liver enlarged, greatly congested, and pervaded by pigment granules. Spleen enlarged, about its normal size, and filled with dark purplish altered blood or splenic mud, rich in hæmaturia, pigment corpuscles, and altered colored blood corpuscles. Kidneys enlarged and greatly congested. Urinary bladder contained about 5 fluid ounces of red urine, which contained some blood. Brain congested; gray matter of a dark chocolate color from deposit of pigment granules and cells.

### CASE IV.

*Pernicious Malarial Fever.—Sudden Death.*—Peter Vooling; age forty-five; native of Ireland; laborer; entered ward 13, bed 163, January 3, 1884.

On the first day of January, 1883, whilst working on the banks the Mississippi river, was seized with a violent chill which rendered him unconscious. He entered Charity Hospital January 3. Slight jaundice; great muscular prostration; rapid pulse, 125 per minute; temperature of axilla 193°F.; stomach very unstable; great tenderness of epigastrium; tongue very red at edges and coated with dark brown fur in centre. When the epigastric region is pressed the patient complains of great tenderness and pain Bowels constipated; complains of violent pain in the occipital region. Urine scanty and contains a small quantity of albumen.

On the morning of January 5, whilst the patient was attempting to eat a little toast and milk, he was suddenly seized with a violent chill; the hospital steward, upon being called to his bed-side, found the patient in a comatose state, his lips and skin covered with a cold, clammy sweat; pupils contracted; in a short time afterwards the patient died.

### CASE V.

*Pernicious Hæmorrhagic Malarial Fever.*—John Golvin; age twenty-one; native of Ireland; laborer; admitted to ward 14, bed 183, Charity Hospital, October 12, 1882; complained of great pain in head and lumbar region; intellect dull; aroused with difficulty, and then complained of pain in the head and back. Bowels constipated. 12 m., patient in full perspiration, dull and drowsy; 8

o'clock p. m., patient stupid, great tenderness in epigastric region; pupils, dilated; temperature, 101°F.; pulse, 120; respiration, 22; tongue red at tip and edges, heavily coated with brown fur.

October 13th—morning—nurse stated that the patient had fallen out of bed during the night. Pulse, very rapid; respiration, 18; temperature of axilla, 104°F.; intellect dull; great tenderness in epigastric region. Patient had passed small quantities of urine during the night and morning. Evening—temperature, 101°F.; pulse, 96; respiration, 18; great tenderness and pain on pressing the epigastric region; patient very restless.

At 9 o'clock, p. m., the patient vomited black matter resembling coffee grounds. Pulse feeble and gaseous; great capillary congestion. Patient died about 2 o'clock, A.M., October 15, 1882.

*Post-Mortem Examination.*—Lungs, normal. Heart, soft and contained an abnormal amount of fat. Stomach, mucous membrane congested, and the viscus contained a small quantity of dark blood. Liver, spleen and kidneys greatly congested. Liver and spleen of a dark, slate color. The urinary bladder contained a small quantity of urine, which, upon examination, was found to be albuminous.

We conclude from the preceding facts:

1. Pernicious paroxysms frequently occur in patients who are suffering from the prolonged action of the malarial poison, which has already induced profound lesions of the blood, brain, liver and spleen.

2. Pernicious malarial fever is frequently characterized by high temperature, which may continue during the most profound coma, even up to death.

3. The phenomena of pernicious (congestive) malarial fever indicate the action of a prompt poison or morbific ferment.— *Virginia Medical Monthly.*

